# Altered Connectivity Pattern of Hubs in Default-Mode Network with Alzheimer's Disease: An Granger Causality Modeling Approach

**DOI:** 10.1371/journal.pone.0025546

**Published:** 2011-10-11

**Authors:** Xiaoyan Miao, Xia Wu, Rui Li, Kewei Chen, Li Yao

**Affiliations:** 1 State Key Laboratory of Cognitive Neuroscience and Learning, Beijing Normal University, Beijing, China; 2 School of Information Science and Technology, Beijing Normal University, Beijing, China; 3 Banner Good Samaritan PET Center, Banner Alzheimer's Institute (BAI), Phoenix, Arizona, United States of America; 4 Beijing Institute Information and Control, Beijing, China; Boston University School of Medicine, United States of America

## Abstract

**Background:**

Evidences from normal subjects suggest that the default-mode network (DMN) has posterior cingulate cortex (PCC), medial prefrontal cortex (MPFC) and inferior parietal cortex (IPC) as its hubs; meanwhile, these DMN nodes are often found to be abnormally recruited in Alzheimer's disease (AD) patients. The issues on how these hubs interact to each other, with the rest nodes of the DMN and the altered pattern of hubs with respect to AD, are still on going discussion for eventual final clarification.

**Principal Findings:**

To address these issues, we investigated the causal influences between any pair of nodes within the DMN using Granger causality analysis and graph-theoretic methods on resting-state fMRI data of 12 young subjects, 16 old normal controls and 15 AD patients respectively. We found that: (1) PCC/MPFC/IPC, especially the PCC, showed the widest and distinctive causal effects on the DMN dynamics in young group; (2) the pattern of DMN hubs was abnormal in AD patients compared to old control: MPFC and IPC had obvious causal interaction disruption with other nodes; the PCC showed outstanding performance for it was the only region having causal relation with all other nodes significantly; (3) the altered relation between hubs and other DMN nodes held potential as a noninvasive biomarker of AD.

**Conclusions:**

Our study, to the best of our knowledge, is the first to support the hub configuration of the DMN from the perspective of causal relationship, and reveal abnormal pattern of the DMN hubs in AD. Findings from young subjects provide additional evidence for the role of PCC/MPFC/IPC acting as hubs in the DMN. Compared to old control, MPFC and IPC lost their roles as hubs owing to the obvious causal interaction disruption, and PCC was preserved as the only hub showing significant causal relations with all other nodes.

## Introduction

The default-mode network (DMN) consists of a set of brain regions showing more increased activity at baseline condition than when performing a wide range of goal-oriented tasks [1]–[3]. Direct evidences from task-free or the resting state studies confirmed these findings [4]–[7]. With these series studies, we now have more solid understanding of the DMN's structure [1], [3], [5], [7]–[10], function [1], [11], [12] and its relevance to diseases [13]–[17]. The core brain regions of DMN, as Buckner and his colleagues suggested, are the medial prefrontal cortex (MPFC), posterior cingulate cortex (PCC), inferior parietal cortex (IPC), inferior temporal cortex (ITC) and (para)hippocampal formation [18].

Of these core DMN brain regions, the PCC, MPFC and IPC play more pivotal roles and are referred as to hubs. Studies converged on that the network shows a configuration centered on these hubs [6], [10], [18]–[22]. For example, both positron emission tomography (PET) and functional magnetic resonance imaging (fMRI) studies have shown that during the resting state, the PCC, MPFC and IPC are more active [6], [19], [20]. Computational analysis of fMRI measured low-frequency blood oxygenation level dependent (BOLD) signal fluctuations advocated the particular stronger presence of spontaneous signal changes in the hub regions than other DMN regions [10]. Consistently, another functional connectivity study demonstrated that hubs had the strongest interregional correlation among themselves and relatively weak correlation with the non-hub regions of the DMN [18]. A subsequent and more detailed whole brain voxel-by-voxel connectivity study showed further the existence of close relationship among hubs with or without the presence of the tasks [21]. Among the DMN hubs, PCC has been suggested to be of special interest since it is the only region directly interacting with all other DMN nodes [22].

The characterization of these DMN hubs is very relevant to the study of aging process itself and brain diseases related to this process. Significant hub specific connectivity/activity/structure abnormity or hypometabolism have been reported in the investigation of normal aging process [23]–[27] and the relevant brain disease [28], [29]. Furthermore, such hub abnormity has been considered as biological markers for a wide spectrum of brain diseases such as schizophrenia [30], autism [31], and attention-deficit/hyperactivity disorder (ADHD) [32]–[34], and, more relevant to this study, the Alzheimer's disease (AD) [15], [21], [35]–[42].

AD is one of the most common neurodegenerative disorders in old population, and generally referred to cognitive function deficits [35]. The disease has been suggested to be associated with disrupted DMN connectivity [15], [36], [37], significant Aβ deposition [21] and notable resting metabolic decline in the DMN hubs and other regions [38], [39]. A well recognized generic risk for AD is the possession of the apolipoprotein ε4 (APOE4) allele [40], [41]. Altered deactivation patterns in DMN have been reported in subjects who were APOE4 carriers, raising the possibility that DMN related abnormalities could serve as a marker for pre-clinical AD studies [42].

While substantial information has been gained about the prominent role of the hubs and the impact of AD on them, the issues on how these hubs interact to each other, with the rest of the DMN regions and their alternations due to AD, require more investigations. Our current study attempts to examine certain aspects of these issues. Especially, we sought to investigate: (1) how DMN hubs interact to each other and to the non-hub regions within DMN; and (2) if these interactions would be altered by AD.

The analytic tool we used in this study is the Granger causality modeling (GCM) technique. First developed and introduced by Ganger in 1969, GCM is one of several methods to infer directional influences among brain regions used in neuroimaging studies [43]–[47]. Compared with structural equation modeling (SEM) and dynamic causal modeling (DCM), GCM is not hypothesis based but data driven. In recent years, it has received a great deal of attention on its application to fMRI data [48]–[50]. Granger causality analysis in this study was done after identifying these hubs and other DMN core regions using independent component analysis (ICA) [9], [15], [17]. We will also discuss the possible definition and use of the Granger causal analysis based biomarker and its sensitivity and specificity in distinguishing AD from old control. Using data from 12 normal young subjects, 16 normal old subjects and 15 AD patients, we found that: (1) there is distinctive causal interaction with the hubs in the DMN in young group, (2) the connectivity pattern of cortical hubs is altered in AD compared to old group, and (3) the alteration holds the potential to serve as a noninvasive biomarker of AD.

## Results

### The Spatial Pattern of DMN in Normal Young Subjects

The spatial pattern of DMN in 12 normal young subjects was detected by using group ICA together with subsequent one sample *t*-test and *p* = 0.05, FDR. The DMN in young subjects included PCC, MPFC, lIPC, rIPC, lITC, rITC, lHC, rHC. Further details on the brain regions in this group have been published in our recent study [51], where the details could be referred.

### The Spatial Pattern of DMN in Normal Controls and AD Subjects

The spatial patterns of DMN in 16 old and 15 AD subjects were each detected using the same approach as for the young group. The DMN in old group included PCC, MPFC, lIPC, rIPC, lITC, rITC, lHC, and rHC. In order to have eight nodes in DMN in the AD patient group as in the old normal group, the left and right HC in the AD group were defined with more lenient threshold of *p* = 0.1 as no voxel survived at *p* = 0.05, FDR. The DMN maps and the between-group DMN difference of the same dataset were previously examined in another separate study [37].

### The Granger Causality DMN results in the Normal Young Subjects

**Figure 1:**
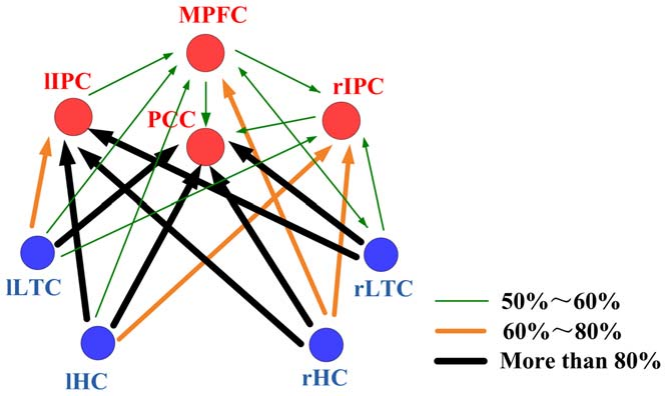
Granger Causality Pattern of the DMN hubs in the young subjects. **DMN** Hubs are colored in red and the non-hub regions in blue. Line width and color indicate the proportion of subjects showing significant causal relationship in this direction (*p* = 0.05). The thickest black lines represent more than 80% subjects showing significant causal influence in this direction, the saffron lines represent 60%∼80%, and the green dashed lines represent 50%∼60%.

**Fig. 1** depicts the Granger causality results of the DMN in normal young group calculated by Granger causality analysis. The arrows pointed toward the nodes (brain regions) that were directionally influenced by the originating ones. Line width and color indicated the proportion of subjects showing significant causal relationship (*p* = 0.05). PCC/MPFC/IPC, especially the PCC, showed the widest and significant casual relationship with all other regions. PCC was the only DMN node that merely received causal influence from other regions. ITC and HC, which both strongly connected with PCC/MPFC/IPC, were not connected with each other directly.

### The Granger Causality DMN results in the Normal Aging and AD Subjects

**Figure 2:**
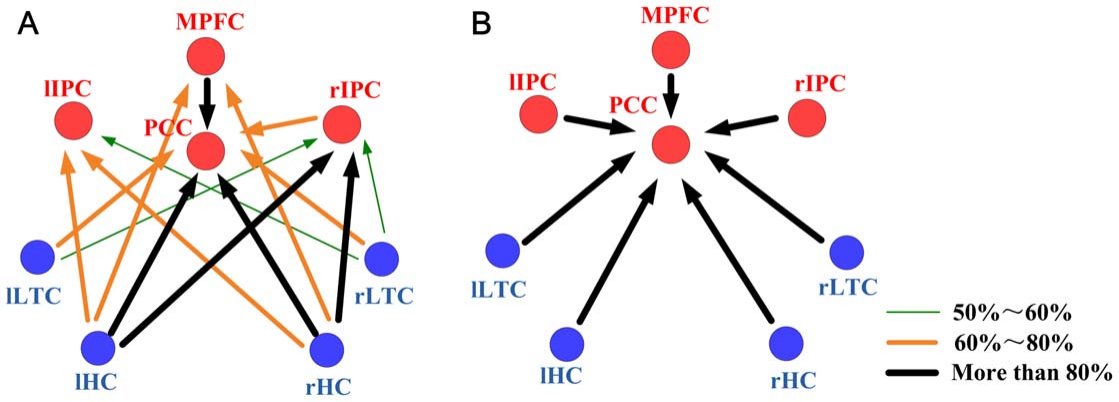
Granger Causality Pattern of the DMN hubs in the normal aging and AD subjects respectively. In the old group (A) and AD (B) group, **DMN** Hubs are colored in red and the non-hub regions in blue. Line width and color indicate the proportion of subjects showing significant causal relationship in this direction (*p* = 0.05). The thickest black lines represent more than 80% subjects showing significant causal influence in this direction, the saffron lines represent 60%∼80%, and the green dashed lines represent 50%∼60%.

Compared to old group, AD patients showed obvious causal interaction attenuation between MPFC and IPC. These two regions also revealed attenuated causal relationship with ITC and HC. Interestingly, we note that the PCC was the only node that had causal relation with all other DMN regions and, again, merely received causal influence from others (**Fig. 2**).

### Altered Relation between Hub and non-Hub Nodes in AD Subjects

**Figure 3:**
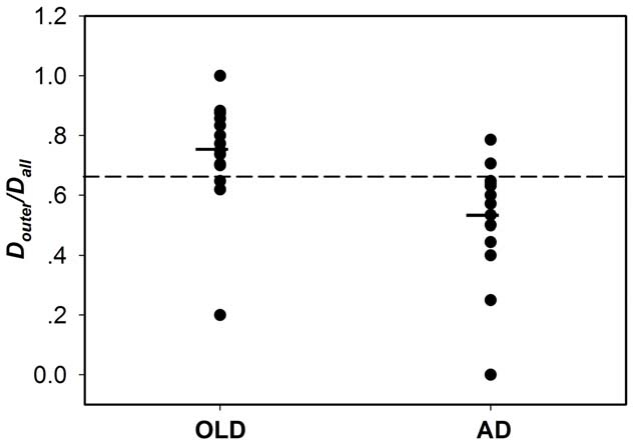
Individual scores for D*_outer_*/D*_all_*. The group means were significantly different in: (D*_outer_*/D*_all_*)_old_>(D*_outer_*/D*_all_*)_AD_ (one-way ANOVA test, *p*<0.05) .The horizontal line indicates a cutoff point of 0.647 by ROC analysis where13 of 16 elderly subjects and 13 of 15 AD patients are correctly categorized.

**Fig. 3** showed the scattergram of D*_outer_*/D*_all_*. Two sample independent test showed that (D*_outer_*/D*_all_*)_old_>(D*_outer_*/D*_all_*)_AD_ (*p* = 0.0015, one-tailed). Examining over all difference among the old and AD subject groups, post-hoc, the group means were significantly different between the old and AD groups using the one-way analysis of variance (ANOVA) test (*p*<0.05). The best cutoff between normal old control and AD, determined by receiver operational characteristic (ROC) approach, was marked in the same scatter plot as a horizontal bar. The cutoff point (0.647) at which 13 of 16 old subjects and 13 of 15 AD patients were correctly categorized yields 81.25% specificity and 80.00% sensitivity.

## Discussion

To the best of our knowledge, this is the first study investigating the configuration of hubs within DMN from the perspective of causal relationship with the use of Granger causality modeling for old normal and AD groups as well as young subjects. Though it is of exploratory nature, the young group was found the strongest causal influences with PCC, MPFC and IPC, and they were directly interacted with virtually all other DMN nodes. More importantly, we found an abnormal pattern of DMN hubs in AD patients compared to old subjects. In addition to these findings, an attempt was made to construct a Granger causal analysis based index to reflect the changes due to AD objectively and quantitatively. Our understandings of the implications of these findings are discussed below.

### Hubs of DMN Revealed in Normal Young Subjects

Researchers have discussed intensively the so called DMN hub regions [6], [18]–[21]. As a whole, the function of DMN has been considered to associate with self referential mental activity [52], stimulus-independent thoughts [53], and monitoring the environment [54] among others. The PCC, MPFC and IPC hub regions play central roles in connecting the DMN brain regions for transmitting information under the mentioned cognitive processes, or optimize the connectivity pattern of DMN to reduce cost of wiring and resources [55]. Reporting that PCC, MPFC and IPC have the widest and distinctive causal relationship with all other regions in the normal young subjects (**Fig. 1**), our study provided additional evidence for the hub central roles.

Of the hub regions, PCC is the most noticeable one since it is the region that has the greatest causal effect relationship with others among the hubs (**Fig. 1**). Earlier PET study showed the highest metabolic activity of the PCC than all other regions during the resting state [56], and recent resting fMRI studies found the PCC was with the most significant functional connectivity in the DMN [3], [18]. Consistently, findings from this current study demonstrated again that PCC is crucial for the function of DMN.

In addition, it is worth to note a new finding in our research is that PCC is the only region which merely received influence from other regions. PCC may be the region to which information converging for the functional cooperation in the DMN. Many studies have shown spontaneous activity of brain in resting state is organized in several specific functional anatomical networks including DMN. However, how these neuronal assemblies communicate and work side by side is still unknown. In lack of any direct evidence, we speculate that the PCC probably works for communication between the DMN and other resting-state networks (RSNs), and hubs like the PCC for DMN may also exist in these RSNs for communication between the networks. Regardless, the dominant role and convergence characteristic of the PCC provide additional insights to elucidate why PCC is generally and obviously abnormal in multiple brain disorders [30], [57]–[59].

### Altered Pattern of Hubs in DMN in AD Subjects

Compared to old subjects, DMN hub connectivity pattern altered in AD group are: 1) the obvious attenuation of causal relation between MPFC/IPC and other nodes and, 2) PCC as the only hub node having causal influence from other DMN regions (**Fig. 2**).

AD is the most common form of dementia which leads to decline in cognitive functioning. The effects of disconnection between cerebral areas on these cognitive functioning such as working memory impairments [60], attention and executive deficits [60] have been explored. Related to these cognitive functions, the reported disconnection were with frontal cortex, parietal cortex, medial temporal cortex and hippocampus formation [60], [61]. For the resting state, studies also showed disrupt functional connectivity in AD patients [15], [35], [62]. IPC has even been suggested to be an important biomarker of AD because it's distinct functional disconnection with frontal cortex [36]. Duara and his colleagues found hypometabolism in frontal and parietal regions in mild and moderate AD patients [63]. All these evidences support the hypothesis that AD includes a disconnection syndrome. Consistent with these previous findings, our results advocated that the disease is correlated with obvious attenuation of causal effects within DMN, and especially the role of MPFC and IPC. The attenuation with these two hubs, we speculate, may be major source of the extensive disruption. The distinct disrupted causal interaction may offer certain perspective of the disease.

PCC is worth special attention for it is the only region having causal relationship with all other nodes in AD subjects while it is the most noticeable hub showing the widest and greatest cause relationship with others nodes in young normal subjects (we note, however, no formal statistical test was performed to examine such differences due to the fact that the MRI data for young subjects were acquired from a different scanner). PCC was under investigation for its various alterations due to the disease [15], [64]–[67].

Although we found no significant greater head movements in AD patients as compared to older normal controls, we are interested in further studying the effects of head motion on the effective connectivity in future separate investigation.

### The Biomarker Characterizing the Disease of AD

We reported D*_outer_*/D*_all_* as an index to characterize the alternation of the relation between hubs as a whole and other DMN nodes due to AD (**Fig. 3**). Compared to old group, significant decrease of D*_outer_*/D*_all_* is shown in AD group. It could be reflective of decreased causal relation between hubs and the other core regions, because of their deterioration or damage caused by the disease. As an index, we found that D*_outer_*/D*_all_* can distinguish individual AD subjects from normal old subjects with reasonable sensitivity of 80.00% and a specificity of 81.25%. More objective assessment of this index's performance will require additional and separate dataset together with cross-validation technique.

In conclusion, we used Granger causality modeling to construct the connectivity pattern of hubs in DMN and examined its abnormalities in AD patients as compared to old normal subjects. The connections for MPFC and IPC as hubs were lost while those for PCC were preserved or even enhanced. Finally, we reported a Granger casualty modeling based index can serve as potential biomarker to distinguish AD patients from old normal subjects with reasonable degree of sensitivity and specificity. Further studies are needed to confirm our findings and to investigate the abnormality of the causal influence of the DMN in the development of AD relative to normal aging, in other types of dementia, and other brain disorders such as ADHD and schizophrenia.

## Materials and Methods

### Subjects and Task

The data acquisition and subjects' demographic characteristics were described. The normal young subjects [51], normal old subjects and AD patients [37] were in previous studies. Briefly, twelve normal young subjects (five males) aged 17 to 27 years old (Mean ± SD: 21±3.4 years old), 16 normal old subjects (seven males) aged 47 to 79 years old (65±9.20), MMSE: 29 (range: 27–30), and fifteen patients (six males) with AD aged 53 to79 years old (64±8.27), average MMSE: 12 (range: 0–20) were scanned during rest state. Five of the 15 AD had a Clinical Dementia Rating (CDR) score of 1 and 6 had CDR 2. The remaining 4 patients had CDR 3. Patients with CDR scores of 1, 2 or 3 were considered to be mild, moderate or severe [68]. Subjects were instructed simply to keep their eyes closed and not to think of anything in particular during the resting-state fMRI scans sessions. Young subjects were recruited and scanned at Beijing Normal University. The purpose of the study was explained to the young participants and each of them gave written informed consent approved by the Research Ethics Committee of the State Key Laboratory of Cognitive Neuroscience and Learning, Beijing Normal University (BNU), prior to the experiment. Old subjects and AD patients were recruited and scanned at Beijing Tiantan Hospital and the purpose of the study was explained to the participants and/or caregivers. All Tiantan Hospital participants themselves and/or their caregivers gave written informed consent approved by the Tiantan Hospital institutional review board before the experiment. The AD patients were free of other diseases and the normal controls were free of any known medical, neurological, and psychiatric disorders. No need for sedation was evidenced the coregistration results (maximal 1.5 mm and1.5^0^ estimated head movements for all subjects) and visual examination. The high resolution volumetric MRI scans were read clinically to exclude patients with evidence of a stroke or any other focal pathology.

### Imaging Methods

#### Beijing Normal University Data

Brain scans were performed at the MRI Center of Beijing Normal University using a 3-T Siemens whole-body MRI scanner. High-resolution structural images were acquired for anatomic reference (repetition time (TR), 2530 ms; Echo time (TE), 3.39 ms; flip angle (FA), 7°; voxel size, 1×1.33×1 mm^3^). Functional images were acquired with T2*-weighted echo planar imaging (EPI) sequences (TR, 2000 ms; TE, 30 ms; FA, 90°; voxel size, 3.13×3.13×3.6 mm^3^; field of view, 256×256 mm^2^ ; 300 time points).

#### Tiantan Hospital Data

Subjects were scanned on 3-Tesla Siemens whole-body MRI system at Tiantan Hospital in Beijing, China. The following parameters were used: structural images (TR, 2100 ms; TE, 3.25 ms; FA,10°; voxel size,1×1×6 mm^3^); functional images (TR, 2000 ms; TE, 30 ms; FA, 85°; voxel size 4×4×6 mm^3^, field of view, 256×256 mm^2^; 250 time points).

### Preprocessing

The same preprocessing, group ICA and selection of the DMN component were the same as in a previous study [37]. For completeness, here are brief descriptions. The fMRI data were preprocessed by using Statistical Parametric Mapping (SPM2, www.fil.ion.ucl.ac.uk/spm). The first 5 functional image acquisitions of each subject were discarded for the possible instability of the initial signal. For each subject, the remaining functional images were realigned to the first volume for possible head movements, corrected for slice-dependent time shifts, spatially normalized to the Montreal neurological institute (MNI) space by individual T1 anatomical image which had been coregistered to the mean functional image after the motion correction, and smoothed by a Gaussian kernel with a full width at half maximum of 8 mm. In the end, the image series were detrended and temporally band-pass filtered (0.01 Hz<*f*<0.1 Hz) to remove linear trends and high-frequency noise using REST (http://restfmri.net).

### Independent Component Analysis

Preprocessed data from all subjects were decomposed into independent components using the GIFT software. The minimum description length (MDL) criterion was used to determine the optimal number of components. 37 components for the group of young subjects, 55 components for the normal old and 59 components for the AD group were determined for next principle component analysis (PCA). In the first round of PCA, the data for each individual subject were dimension-reduced to the optimal number temporally. After concatenation across subjects within group, the dimensions were again reduced to the optimal number via the second round of PCA. Then the data were separated by ICA using the Extended Infomax algorithm [69]. After ICA separation, the mean independent components (ICs) and the corresponding mean time courses over all the subjects were used for the back-reconstruction of the ICs and the time courses for each individual subject [69].

### Selection of the Best-Fit Component

The DMN was identified by template goodness fit and visual inspection [37]. To do this, a DMN template was developed based on a dataset of regions reported previously [70]. Each region in the template was a sphere with a radius of 5 mm (varying size of the sphere had no effect for the component identification). To determine the DMN among a number of independent components for a subject, the average intensity over voxels within each of the spheres minus that over voxels outside all spheres was for each component. Finally, the component that had the best-fit was designated as DMN for this subject. For group analysis, one sample *t*-test (height threshold: False Discovery Rate, *p* = 0.05, FDR, extend threshold: *k* = 10 voxels) for each of the 3 groups was used to determine the group DMN [71].

### Definition of Group Specific DMN Core Regions and Data Extraction

For normal young and old subjects, we identified eight DMN ROIs based on their respective group DMN map. Each of the eight ROIs was defined as the intersection of voxels exceeded the threshold in the one sample *t*-test DMN map (*p* = 0.05, FDR) and a sphere with a radius of 10 mm. The coordinate of the sphere center was selected as the coordinate of the voxel in each of the DMN regions showing highest statistical significance (*p* = 0.05, FDR) in the group DMN map by xjView toolbox for SPM (http://people.hnl.bcm.tmc.edu/cuixu/xjView/). For the AD group, the ROIs of the left and right HC were defined as the same interaction but with *p* = 0.1, FDR since no voxel survived at *p* = 0.05. Time series for each region were then extracted from each subject. Global signal was removed from the time series to minimize the variance contributed by physiological artifacts and scanner drift [72]. The average time courses for each core region were input to the Granger causality analysis.

### Granger Causality Modeling Analysis

Granger causality modeling is an approach to explore dynamic causal relationship between two time series [43], [44]. In the neuroimaging studies, it was first applied to electroencephalography (EEG) and magneto encephalography (MEG) data [45]–[47], and later to fMRI data [48]–[50]. For completeness, a brief introduction of the Granger procedure is provided here.

For two given fMRI time series x(n) and y(n), x(n) is called Granger causing y(n) if the past information of x(n) can improve the prediction of the current value of y(n). The Granger causal relation between the two series is often estimated by vector autoregressive (VAR) modeling. Granger causality can evaluate the linear direct influence from x(n) to y(n) (F*_XtoY_*), the linear direct influence from y(n) to x(n) (F*_YtoX_*) and the instantaneous influence between x(n) and y(n) (F*_X·Y_*). The instantaneous influence F*_X·Y_* essentially reflects partial correlation that cannot be assigned to influence in a certain direction purely from temporal information [49]. Thus, for the interest of directed influences, we only estimated and focused on F*_XtoY_* and F*_YtoX_*.

In this study, Granger causality analysis was performed using in-house developed MATLAB code. F*_XtoY_* and F*_YtoX_* were calculated based on VAR model using Fast Orthogonal Searching (FOS) algorithm [73]. The order of the VAR was selected as 5 because the delay between regions was nearly 8s [73]. Then, 1000 surrogate data were generated by Iterated Amplitude Adjusted Fourier-transformed (IAAFT) surrogate to test the significance of the Granger causality (*p* = 0.05) [74]. In order to extract information on the Granger causality analysis better, causal relations between DMN nodes were represented as directed graphs. To explore the use of Granger causality based index to differentiate AD from normal old subjects, D*_out_* of a node was defined as the number of afferent connections from the node to any others, and D*_in_* of a node as the number of efferent connections from any of the other nodes to the node [75], [76].

We then defined D*_outer_* as the sum of the D*_out_* and D*_in_* between any hub and any non-hub nodes of the DMN for a given subject [77]. In addition, we defined D*_all_* as the sum of D*_out_* and D*_in_* between any two DMN regions, hub or non-hub. Finally, we calculated D*_outer_*/D*_all_*, which was used to describe the ability of hubs to communicate with these non-hub nodes relative to the whole DMN and was used as the DMN hub index to categorize AD and old normal controls using ROC analysis described below.

### Receiver Operational Characteristic (ROC) Analysis

The ROC analysis was performed to obtain the optimal cut-off value of D*_outer_*/D*_all_* in distinguishing AD patients from old control. ROC analysis has been commonly used to characterize the sensitivity and specificity of a biomarker for distinguishing a patient group from another group such as normal controls in this study [78]. Under ROC analysis, sensitivity is the true-positive rate, indicating the proportion of patients whose biomarker test is positive. On the other hand, specificity is the true-negative rate, indicating the proportion of non-disease subjects which are correctly identified. As stated above, the present study used ROC curve analysis to determine the threshold with which the sensitivity and specificity are optimal for the DMN D*_outer_*/D*_all_* index as a biomarker.

## References

[1] Raichle ME, MacLeod AM, Snyder AZ, Powers WJ, Gusnard DA (2001). A default mode of brain function.. Proc Natl Acad Sci U S A.

[2] Raichle ME, Snyder AZ (2007). A default mode of brain function: a brief history of an evolving idea.. Neuroimage.

[3] Greicius MD, Krasnow B, Reiss AL, Menon V (2003). Functional connectivity in the resting brain: a network analysis of the default mode hypothesis.. Proc Natl Acad Sci U S A.

[4] Shulman GL, Fiez JA, Corbetta M, Buckner RL, Miezin FM (1997). Common blood flow changes across visual tasks: II. Decreases in cerebral cortex.. Journal of Cognitive Neuroscience.

[5] Mazoyer B, Zago L, Mellet E, Bricogne S, Etard O (2001). Cortical networks for working memory and executive functions sustain the conscious resting state in man.. Brain Res Bull.

[6] Shannon BJ (2006).

[7] Fox MD, Snyder AZ, Vincent JL, Corbetta M, Van Essen DC (2005). The human brain is intrinsically organized into dynamic, anticorrelated functional networks.. Proc Natl Acad Sci U S A.

[8] Vincent JL, Snyder AZ, Fox MD, Shannon BJ, Andrews JR (2006). Coherent spontaneous activity identifies a hippocampal-parietal memory network.. J Neurophysiol.

[9] Damoiseaux JS, Rombouts SA, Barkhof F, Scheltens P, Stam CJ (2006). Consistent resting-state networks across healthy subjects.. Proc Natl Acad Sci U S A.

[10] Fransson P (2005). Spontaneous low-frequency BOLD signal fluctuations: an fMRI investigation of the resting-state default mode of brain function hypothesis.. Hum Brain Mapp.

[11] Gilbert SJ, Dumontheil I, Simons JS, Frith CD, Burgess PW (2007). Comment on “Wandering minds: the default network and stimulus-independent thought”.. Science.

[12] Svoboda E, McKinnon MC, Levine B (2006). The functional neuroanatomy of autobiographical memory: a meta-analysis.. Neuropsychologia.

[13] Celone KA, Calhoun VD, Dickerson BC, Atri A, Chua EF (2006). Alterations in memory networks in mild cognitive impairment and Alzheimer's disease: an independent component analysis.. J Neurosci.

[14] Cherkassky VL, Kana RK, Keller TA, Just MA (2006). Functional connectivity in a baseline resting-state network in autism.. Neuroreport.

[15] Greicius MD, Srivastava G, Reiss AL, Menon V (2004). Default-mode network activity distinguishes Alzheimer's disease from healthy aging: evidence from functional MRI.. Proc Natl Acad Sci U S A.

[16] Rombouts SA, Barkhof F, Goekoop R, Stam CJ, Scheltens P (2005). Altered resting state networks in mild cognitive impairment and mild Alzheimer's disease: an fMRI study.. Hum Brain Mapp.

[17] Sorg C, Riedl V, Muhlau M, Calhoun VD, Eichele T (2007). Selective changes of resting-state networks in individuals at risk for Alzheimer's disease.. Proc Natl Acad Sci U S A.

[18] Buckner RL, Andrews-Hanna JR, Schacter DL (2008). The brain's default network: anatomy, function, and relevance to disease.. Ann N Y Acad Sci.

[19] Gold BT, Buckner RL (2002). Common prefrontal regions coactivate with dissociable posterior regions during controlled semantic and phonological tasks.. Neuron.

[20] Lustig C, Buckner RL (2004). Preserved neural correlates of priming in old age and dementia.. Neuron.

[21] Buckner RL, Sepulcre J, Talukdar T, Krienen FM, Liu H (2009). Cortical hubs revealed by intrinsic functional connectivity: mapping, assessment of stability, and relation to Alzheimer's disease.. J Neurosci.

[22] Fransson P, Marrelec G (2008). The precuneus/posterior cingulate cortex plays a pivotal role in the default mode network: Evidence from a partial correlation network analysis.. Neuroimage.

[23] Herholz K, Salmon E, Perani D, Baron JC, Holthoff V (2002). Discrimination between Alzheimer dementia and controls by automated analysis of multicenter FDG PET.. Neuroimage.

[24] Pardo JV, Lee JT, Sheikh SA, Surerus-Johnson C, Shah H (2007). Where the brain grows old: decline in anterior cingulate and medial prefrontal function with normal aging.. Neuroimage.

[25] Goldberg S, Smith GS, Barnes A, Ma Y, Kramer E (2004). Serotonin modulation of cerebral glucose metabolism in normal aging.. Neurobiol Aging.

[26] Grady CL, Springer MV, Hongwanishkul D, McIntosh AR, Winocur G (2006). Age-related changes in brain activity across the adult lifespan.. J Cogn Neurosci.

[27] Lustig C, Snyder AZ, Bhakta M, O'Brien KC, McAvoy M (2003). Functional deactivations: change with age and dementia of the Alzheimer type.. Proc Natl Acad Sci U S A.

[28] Sambataro F, Murty VP, Callicott JH, Tan HY, Das S (2008). Age-related alterations in default mode network: impact on working memory performance.. Neurobiol Aging.

[29] Andrews-Hanna JR, Snyder AZ, Vincent JL, Lustig C, Head D (2007). Disruption of large-scale brain systems in advanced aging.. Neuron.

[30] Garrity AG, Pearlson GD, McKiernan K, Lloyd D, Kiehl KA (2007). Aberrant “default mode” functional connectivity in schizophrenia.. American Journal of Psychiatry.

[31] Kennedy DP, Redcay E, Courchesne E (2006). Failing to deactivate: resting functional abnormalities in autism.. Proc Natl Acad Sci U S A.

[32] Castellanos FX, Margulies DS, Kelly C, Uddin LQ, Ghaffari M (2008). Cingulate-precuneus interactions: a new locus of dysfunction in adult attention-deficit/hyperactivity disorder.. Biol Psychiatry.

[33] Mulas F, Capilla A, Fernandez S, Etchepareborda MC, Campo P (2006). Shifting-related brain magnetic activity in attention-deficit/hyperactivity disorder.. Biol Psychiatry.

[34] Durston S, Mulder M, Casey BJ, Ziermans T, van Engeland H (2006). Activation in ventral prefrontal cortex is sensitive to genetic vulnerability for attention-deficit hyperactivity disorder.. Biol Psychiatry.

[35] Delbeuck X, Van der Linden M, Collette F (2003). Alzheimer's disease as a disconnection syndrome?. Neuropsychol Rev.

[36] Wang K, Liang M, Wang L, Tian L, Zhang X (2007). Altered functional connectivity in early Alzheimer's disease: a resting-state fMRI study.. Hum Brain Mapp.

[37] Wu X, Li R, Fleisher AS, Reiman EM, Guan X (2011). Altered default mode network connectivity in alzheimer's disease—A resting functional MRI and bayesian network study.. Human brain mapping.

[38] Minoshima S, Giordani B, Berent S, Frey KA, Foster NL (1997). Metabolic reduction in the posterior cingulate cortex in very early Alzheimer's disease.. Ann Neurol.

[39] Alexander GE, Chen K, Pietrini P, Rapoport SI, Reiman EM (2002). Longitudinal PET Evaluation of Cerebral Metabolic Decline in Dementia: A Potential Outcome Measure in Alzheimer's Disease Treatment Studies.. Am J Psychiatry.

[40] Reiman EM, Caselli RJ, Yun LS, Chen K, Bandy D (1996). Preclinical evidence of Alzheimer's disease in persons homozygous for the ε4 allele for apolipoprotein E.. New England Journal of Medicine.

[41] Reiman EM, Chen K, Alexander GE, Caselli RJ, Bandy D (2004). Functional brain abnormalities in young adults at genetic risk for late-onset Alzheimer's dementia.. Proceedings of the National Academy of Sciences of the United States of America.

[42] Fleisher AS, Sherzai A, Taylor C, Langbaum J, Chen K (2009). Resting-state BOLD networks versus task-associated functional MRI for distinguishing Alzheimer's disease risk groups.. Neuroimage.

[43] Granger CWJ (1969). Investigating causal relations by econometric models and cross-spectral methods.. Econometrica.

[44] Granger CWJ (1980). Testing for causality: A personal viewpoint.. Journal of Economic Dynamics and Control.

[45] Bakardjian H, Babiloni F, Cichocki A, Cincotti F, Marciani MG (2006). P31.3 On the estimation of causality between cortical spatial patterns during voluntary movements in normal subjects by using independent component analysis.. Clinical Neurophysiology.

[46] Brovelli A, Ding M, Ledberg A, Chen Y, Nakamura R (2004). Beta oscillations in a large-scale sensorimotor cortical network: directional influences revealed by Granger causality.. Proc Natl Acad Sci U S A.

[47] Kaminski M, Ding M, Truccolo WA, Bressler SL (2001). Evaluating causal relations in neural systems: granger causality, directed transfer function and statistical assessment of significance.. Biol Cybern.

[48] Goebel R, Roebroeck A, Kim DS, Formisano E (2003). Investigating directed cortical interactions in time-resolved fMRI data using vector autoregressive modeling and Granger causality mapping.. Magn Reson Imaging.

[49] Roebroeck A, Formisano E, Goebel R (2005). Mapping directed influence over the brain using Granger causality and fMRI.. Neuroimage.

[50] Gao Q, Chen H, Gong Q (2008). Evaluation of the effective connectivity of the dominant primary motor cortex during bimanual movement using Granger causality.. Neuroscience Letters.

[51] Li R, Chen K, Fleisher AS, Reiman EM, Yao L (2011). Large-scale directional connections among multi resting-state neural networks in human brain: A functional MRI and Bayesian network modeling study.. NeuroImage.

[52] Wicker B, Ruby P, Royet JP, Fonlupt P (2003). A relation between rest and the self in the brain?. Brain Res Brain Res Rev.

[53] Mason MF, Norton MI, Van Horn JD, Wegner DM, Grafton ST (2007). Wandering minds: the default network and stimulus-independent thought.. Science.

[54] Gilbert DT, Wilson TD (2007). Prospection: experiencing the future.. Science.

[55] Bassett DS, Bullmore E (2006). Small-world brain networks.. Neuroscientist.

[56] Gusnard DA, Raichle ME (2001). Searching for a baseline: functional imaging and the resting human brain.. Nat Rev Neurosci.

[57] Rubia K, Smith AB, Halari R, Matsukura F, Mohammad M (2009). Disorder-specific dissociation of orbitofrontal dysfunction in boys with pure conduct disorder during reward and ventrolateral prefrontal dysfunction in boys with pure ADHD during sustained attention.. Am J Psychiatry.

[58] Haznedar MM, Buchsbaum MS, Wei TC, Hof PR, Cartwright C (2000). Limbic circuitry in patients with autism spectrum disorders studied with positron emission tomography and magnetic resonance imaging.. Am J Psychiatry.

[59] Benson DF, Kuhl DE, Hawkins RA, Phelps ME, Cummings JL (1983). The fluorodeoxyglucose 18F scan in Alzheimer's disease and multi-infarct dementia.. Arch Neurol.

[60] Collette F, Salmon E, Van der Linden M, Chicherio C, Belleville S (1999). Regional brain activity during tasks devoted to the central executive of working memory.. Cognitive Brain Research.

[61] Perry RJ, Hodges JR (1999). Attention and executive deficits in Alzheimer's disease. A critical review.. Brain.

[62] Allen G, Barnard H, McColl R, Hester AL, Fields JA (2007). Reduced hippocampal functional connectivity in Alzheimer disease.. Archives of neurology.

[63] Maxim V, Sendur L, Fadili J, Suckling J, Gould R (2005). Fractional Gaussian noise, functional MRI and Alzheimer's disease.. Neuroimage.

[64] Chetelat G, Desgranges B, De La Sayette V, Viader F, Eustache F (2003). Mild cognitive impairment.. Neurology.

[65] Hirao K, Ohnishi T, Hirata Y, Yamashita F, Mori T (2005). The prediction of rapid conversion to Alzheimer's disease in mild cognitive impairment using regional cerebral blood flow SPECT.. Neuroimage.

[66] Bai F, Zhang Z, Watson DR, Yu H, Shi Y (2009). Abnormal functional connectivity of hippocampus during episodic memory retrieval processing network in amnestic mild cognitive impairment.. Biological psychiatry.

[67] Zhang HY, Wang SJ, Xing J, Liu B, Ma ZL (2009). Detection of PCC functional connectivity characteristics in resting-state fMRI in mild Alzheimer's disease.. Behavioural brain research.

[68] Morris JC (1993). The Clinical Dementia Rating (CDR): current version and scoring rules.. Neurology.

[69] Lee TW, Girolami M, Sejnowski TJ (1999). Independent component analysis using an extended infomax algorithm for mixed subgaussian and supergaussian sources.. Neural Comput.

[70] Mantini D, Perrucci MG, Del Gratta C, Romani GL, Corbetta M (2007). Electrophysiological signatures of resting state networks in the human brain.. Proc Natl Acad Sci U S A.

[71] Calhoun VD, Adali T, McGinty VB, Pekar JJ, Watson TD (2001). fMRI activation in a visual-perception task: network of areas detected using the general linear model and independent components analysis.. Neuroimage.

[72] Macey PM, Macey KE, Kumar R, Harper RM (2004). A method for removal of global effects from fMRI time series.. Neuroimage.

[73] Bagarinao E, Sato S (2002). Algorithm for vector autoregressive model parameter estimation using an orthogonalization procedure.. Ann Biomed Eng.

[74] Schreiber T, Schmitz A (2000). Surrogate time series.. Physica D: Nonlinear Phenomena.

[75] Seth AK (2005). Causal connectivity of evolved neural networks during behavior.. Network: Computation in Neural Systems.

[76] Sridharan D, Levitin DJ, Menon V (2008). A critical role for the right fronto-insular cortex in switching between central-executive and default-mode networks.. Proceedings of the National Academy of Sciences.

[77] Uddin LQ, Kelly AM, Biswal BB, Xavier Castellanos F, Milham MP (2009). Functional connectivity of default mode network components: correlation, anticorrelation, and causality.. Hum Brain Mapp.

[78] Zweig MH, Campbell G (1993). Receiver-operating characteristic (ROC) plots: a fundamental evaluation tool in clinical medicine [published erratum appears in Clin Chem 1993 Aug; 39 (8): 1589].. Clinical chemistry.

